# Gelatin methacryloyl hydrogel as a potential scaffold for cell transplantation for Huntington’s disease

**DOI:** 10.1093/braincomms/fcag295

**Published:** 2026-07-27

**Authors:** Linh Nguyen, Amy McCaughey-Chapman, Bronwen Connor

**Affiliations:** Department of Pharmacology and Clinical Pharmacology, Centre for Brain Research, School of Medical Science, Faculty of Medical and Health Sciences, University of Auckland, Auckland 1023, New Zealand; Department of Pharmacology and Clinical Pharmacology, Centre for Brain Research, School of Medical Science, Faculty of Medical and Health Sciences, University of Auckland, Auckland 1023, New Zealand; Department of Pharmacology and Clinical Pharmacology, Centre for Brain Research, School of Medical Science, Faculty of Medical and Health Sciences, University of Auckland, Auckland 1023, New Zealand

**Keywords:** cell transplantation, direct reprogramming, Huntington’s disease, GelMA hydrogel

## Abstract

Cell replacement therapy is a promising strategy for treating Huntington’s disease. Advances in cell reprogramming now allow the generation of clinically relevant cells; however, conventional delivery methods can compromise long-term cell survival and maturation. Hydrogels have emerged as supportive three-dimensional scaffolds that better mimic the *in vivo* environment. This study examined whether encapsulating human-induced lateral ganglionic eminence precursor cells (hiLGEPs) in gelatin methacryloyl (GelMA) hydrogel enhanced differentiation after transplantation into the striatum of the quinolinic acid lesion rat model of Huntington’s disease. hiLGEPs were delivered either in cell culture medium (*n* = 5) or in a pre-crosslinked GelMA solution (*n* = 4) prior to transplantation. GelMA was polymerized *in situ* using a visible-light optical fibre system, and grafts were assessed 4 weeks later. Transplanted cells survived in all animals. Notably, GelMA-encapsulated hiLGEPs produced significantly more protein gene product 9.5 (PGP9.5^+^) neurons than those delivered in cell culture medium. Transplanted cells in both groups differentiated into microtubule-associated protein 2 (MAP2^+^) and GABA^+^ phenotypes, with no detectable differences in graft volume. These findings provide the first evidence that encapsulating hiLGEPs in GelMA enhances early neuronal differentiation following transplantation, supporting GelMA hydrogel as a promising delivery scaffold for cell replacement therapy in Huntington’s disease.

## Teaser

Nguyen *et al*. report proof-of-concept data demonstrating that transplanting human-induced lateral ganglionic eminence precursor cells encapsulated in gelatin methacryloyl hydrogel in the quinolinic acid lesioned striatum enhances early neuronal differentiation. This provides initial evidence that gelatin methacryloyl hydrogel is a viable biocompatible scaffold to augment cell replacement therapy.

## Graphical Abstract

**Graphical Abstract:**
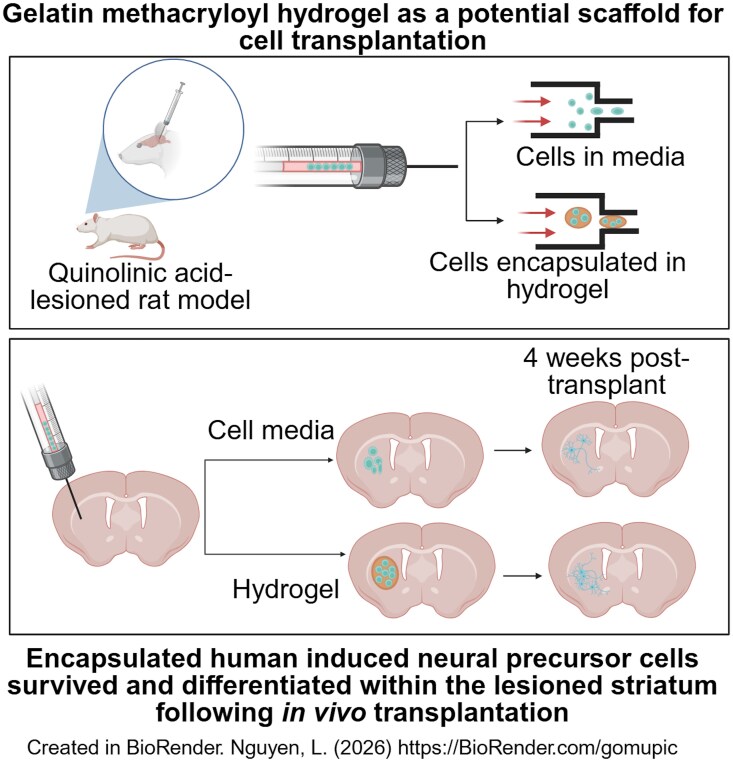

## Introduction

Huntington’s disease is an autosomal dominant neurodegenerative disease by an abnormal expansion of CAG trinucleotide repeats (>36 repeats) on the *huntingtin* gene, producing mutant huntingtin protein that drives progressive degeneration of medium spiny neurons (MSNs) in the striatum.^1-3^ Cell replacement therapy (CRT) is a promising strategy to treat Huntington’s disease by replenishing lost MSNs and restoring neuronal circuit function.^4^ To achieve clinical translation, an ethically viable cell source and optimized delivery strategy are essential.

Cell reprogramming technologies provide an ethically viable human cell source for CRT. We have established a protocol to directly reprogramme adult human dermal fibroblasts (aHDFs) into human-induced lateral ganglionic eminence precursor cells (hiLGEPs) using chemically modified mRNA (cmRNA).^5^ These cells differentiate into TUJ1^+^ and DARPP32^+^ striatal neurons *in vitro* and survive transplantation into the quinolinic acid (QA)–lesioned rat model of Huntington’s disease. Transplanted hiLGEPs differentiated into DARPP32^+^ MSNs and improved motor outcomes 14 weeks post-transplant.^5^

Cell delivery methods are a major determinant of CRT success. Traditional injection of cells in culture medium exposes them to mechanical stress, reducing survival. Biomaterial scaffolds such as hydrogels can provide structural support, reduce shear damage and, by mimicking the extracellular matrix, promote survival and maturation after transplantation.^6^ Gelatin methacryloyl (GelMA) hydrogel is a promising candidate because it is biocompatible, provides a hydrated environment supporting adhesion and survival and offers tuneable mechanical properties.^7-9^ GelMA forms a crosslinked network in the presence of light and photoinitiator.^10^ UV-sensitive initiators are commonly used but may cause DNA damage^11,12^; visible-light chemistries such as ruthenium (Ru) and sodium persulfate (SPS) are therefore a safer alternative.^13,14^ We have previously shown that hiLGEPs encapsulated in GelMA crosslinked with Ru/SPS exhibit enhanced viability and MSN differentiation *in vitro* and *ex vivo.*^7^

This study investigated the ability of directly reprogrammed hiLGEPs to survive and differentiate when encapsulated in visible-light-crosslinking GelMA hydrogel and transplanted into the QA-lesioned rat striatum. We hypothesized that GelMA would provide a structural scaffold that supports cell survival, neuronal differentiation and protection against host tissue responses *in vivo*. An optical fibre system will be utilized to induce GelMA photopolymerisation at the transplant site.

## Materials and methods

### Cell reprogramming

aHDFs (female Caucasian, abdominal tissue) were obtained from Invitrogen and expanded in Dulbecco’s Modified Eagle Medium (Thermo Fisher Scientific) with 10% fetal bovine serum (Thermo Fisher Scientific). At 80–90% confluency, cells were replated and transfected the following day. On Day 0, aHDFs were placed in reprogramming medium containing BrainPhys (Stem Cell Technologies), 1 mM valproic acid (Sigma-Aldrich), 1% penicillin-streptomycin-glutamine (Gibco™), 2% B27™ supplement without vitamin A (Gibco™), 20 ng/mL fibroblast growth factor 2 (Prospec Bio), 20 ng/mL epidermal growth factor (Prospec Bio), 20 µg/mL Heparin (Sigma-Aldrich), 1% N2 supplement (Gibco™), 5 µM Gö6983 (Abcam), 10 µM Y27632 (Abcam) and 10 µM retinoic acid (Sigma). Cells were then transfected by diluting 100 µg/mL lipid nanoparticle cmRNA *SOX2/PAX6* (Ethris GmbH, Munich, Germany) in OptiMEM (Thermo Fisher Scientific), with the final concentration of 1.125 µg/mL of the mix on cells. Twenty-four hours later, the transfection mix was replaced with fresh reprogramming medium which was refreshed every two days until Day 7, when cells were passaged and 25 ng/mL activin A (Prospec Bio) was included in the reprogramming media. After 14 days, cells were designated hiLGEPs. The overall timeline for *in vitro* culture is shown in Fig. 1.

**Figure 1:**
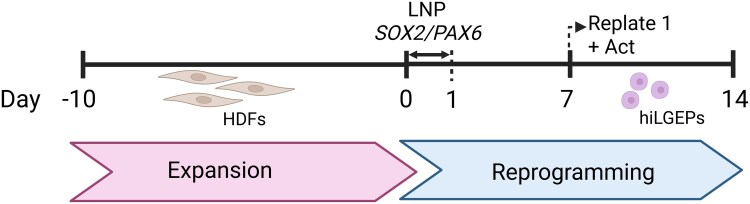
**Schematic diagram demonstrating the direct reprogramming protocol.** Briefly, adult human dermal fibroblasts (aHDFs) were transfected with lipid nanoparticles (LNP) cmRNA *SOX2/PAX6* for reprogramming. At Day 7, activin A was introduced into the media to generate human-induced lateral ganglionic eminence precursor cells (hiLGEPs). Created in BioRender. Nguyen, L. (2026) https://BioRender.com/kfikvzm.

### Optical fibre setup

A mounted neutral-white LED (ThorLabs MNWHL4) was coupled to an implantable optical fibre cannula (200 µm core, 50 mm in length, CFM12L10) via a patch cable (Fig. 2).

**Figure 2:**
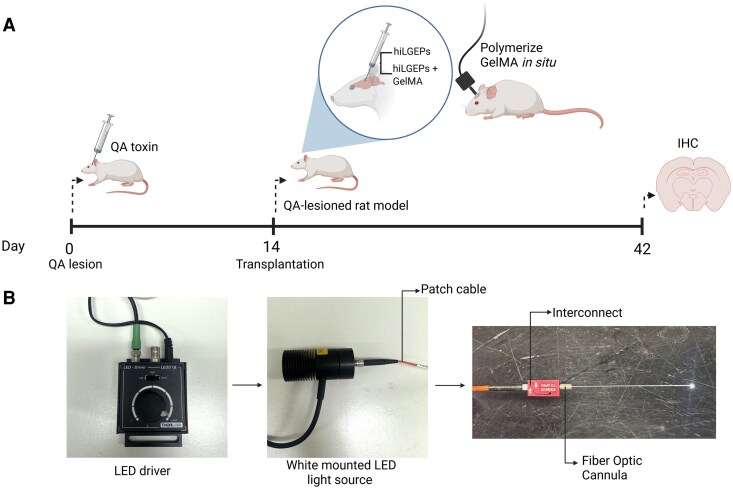
**Cell transplantation methodology.** (**A**) Schematic timeline of hiLGEPs *in vivo* transplantation into the striatum of the QA-lesioned rat model of Huntington’s disease. (**B**) Setup of the optical fibre system. QA, quinolinic acid; hiLGEPs, human-induced lateral ganglionic eminence precursor cells; GelMA, gelatin methacryloyl; IHC, immunohistochemistry. Created in BioRender. Nguyen, L. (2026) https://BioRender.com/5bznpvg.

### Gelatin methacryloyl fabrication

Gelatin (porcine skin, type A, 300 g bloom; Sigma-Aldrich) was dissolved in 0.25 M carbonate buffer, then adjusted to pH 9. Methacrylate anhydride (Sigma-Aldrich) was slowly added into the gelatin A solution (0.05 mL per gram of gelatin) at 40°C with continued stirring, and the solution was allowed to react for 3 h. The solution was dialysed using a 14 kDa molecular weight cut-off dialysis membrane (Membra-Cel®, Viskase) against Milli-Q water at 37°C for 7 days to eliminate unreacted methacrylate. The solution was then centrifuged at 37°C for 15 min, the supernatant collected for freeze drying and the GelMA precursor polymer obtained.

### Gelatin methacryloyl hydrogel preparation

A 20 wt% GelMA polymer solution was prepared in PBS. Photoinitiator stock solutions of 50 mM Ru and 500 mM SPS were prepared separately. Pre-gel solutions were prepared by combining the polymer with Ru, SPS and PBS to generate a 10 wt% GelMA final mixture with 0.5 mM Ru and 5 mM SPS. Polymerization was induced by exposure to visible light for ∼2 min.

### Gelatin methacryloyl hydrogel stiffness

The hydrogel stiffness was determined using atomic force microscopy (AFM, MFP-3D Origin AFM, Asylum Research, USA) to calculate the Young’s modulus, following.^15^ The samples were prepared by dispensing the GelMA solution onto glutaraldehyde-functionalized silicon wafer moulds, then flattened with a hydrophobic coverslip, and immediately functionalized under LED light for 2 min (*n* = 3 hydrogels). The samples were submerged in 1× PBS at room temperature overnight prior to AFM analysis.

The AFM measurements for all samples were conducted in PBS using a cantilever (Novascan, Iowa, USA). The cantilever (spring constant = 0.07 N/m, calibrated by the manufacturer) has a 5 µm diameter spherical silicon nitride tip. The trigger force was measured at 5 nN with a velocity of 2.5 µm/s. The instrumental optical lever sensitivity was calibrated three times in PBS by indenting a glass slide, and the readings were averaged.

For each GelMA sample, three force maps were randomly measured at 10 × 10 force points over an area of 90 µm × 90 µm. The force maps were analysed using Asylum Research Software AR16, and the curve was fitted with the Hertz model to calculate Young’s modulus.

### Swelling test

Approximately 1.2 mL of hydrogel was dispensed into an acrylic mould with a diameter of 2 cm and a thickness of 3 mm, then crosslinked for 5 min with LED light. Gels were left in a 37°C for 48 h, covered in foil and then weighed ($m_{dry}$). The samples were then submerged in 5 mL of 1× PBS for 24 h and weighed ($m_{wet}$). The swelling test was conducted on three hydrogels across three trials. The swelling ratio was calculated using the following formula and averaged across the trials: $Swelling\;ratio=\;\frac{m_{wet-\;m_{dry}}}{m_{dry}}$.

### Animals

Eight-week-old male Sprague–Dawley rats were obtained from the Vernon Jansen Unit at the University of Auckland. At the time of QA lesioning, the rats weighed between 250 and 350 g. All experimental procedures adhered to the University of Auckland Animal Ethics Guidelines, in compliance with the New Zealand Animal Welfare Act 1999 and ARRIVE 2.0 guidelines. Animals were randomly assigned to two treatment groups: (i) hiLGEPs delivered in cell reprogramming medium (*n* = 5) and (ii) hiLGEPs encapsulated in GelMA hydrogel (*n* = 4). True randomization was not used to allocate experimental units. Instead, a systematic allocation method was employed where rats were sequentially assigned to groups in an alternating fashion as they were removed from their cages. The sequence was not hidden from the investigator. Animals were housed four to five per cage in a temperature- and humidity-controlled room on a 12-h light/dark cycle, with *ad libitum* access to food and water and cage enrichment. All rats received subcutaneous injections of cyclosporin A (Sandimmun, 15 mg/kg) three times per week post-surgery.

### Surgical procedures and cell transplantation *in vivo*

Under isoflurane anaesthesia, rats received unilateral QA lesions (400 nL of 50 nM QA) of the striatum 14 days before transplantation (+0.5 mm AP and −2.7 mm ML relative to bregma and −5.0 mm DV from dura). Approximately 250 000 hiLGEPs resuspended either in 4 µL of cell culture medium or GelMA hydrogel were injected at two adjacent sites in the lesioned striatum (2 µL cell suspension/site) at the following stereotaxic coordinates: +0.3 mm AP, −2.5 mm ML and −5.0 mm or −4.0 mm DV. The cell culture medium contained BrainPhys (Stem Cell Technologies), 1% PSG (Gibco™), 2% B27™ supplement without vitamin A (Gibco™), 1% N2 supplement (Gibco™), 10 µM Forskolin (Abcam), 10 µM Y27632 (Abcam) and 30 ng/mL brain-derived neurotrophic factor (Prospec Bio). For GelMA transplants, *in situ* photopolymerisation was carried out by exposing the injection site to LED light for 2 min via the optical fibre. The overall timeline for the *in vivo* transplantation is depicted in Fig. 2. Potential procedural and environmental confounders were controlled in this study. Specifically, cage allocation on the holding racks was randomized, and animals were treated and measured by sequenced randomization.

### Immunohistochemistry

Four weeks post-transplant, animals were transcardially perfused with 4% paraformaldehyde after terminal anaesthesia with sodium pentobarbitone (120 mg/kg, IP) and the brains processed for coronal sectioning using a freezing microtome (SM2010R, Leica). Free-floating sections were stained with antibodies against STEM121 (1:500, Takara Bio), PGP9.5 (1:500, Abcam), MAP2 (1:500, Abcam), GABA (1:500, Invitrogen), GFAP (1:500, DAKO), Iba-1 (1:250, Abcam) and DARPP32 (1:500, Invitrogen). Secondary antibodies and DAPI nuclear counterstain were applied before confocal microscopy. Brain sections were imaged using a Zeiss LSM 800 Airyscan [Biomedical Imaging Research Unit (BIRU), University of Auckland]. To assess graft volume, whole tissue sections with STEM121^+^ grafts were imaged with an Olympus SLIDEVIEW VS200 slide scanner (BIRU), and the area of interest containing the grafts was captured at 20× magnification. Images were acquired using CellSense FV Software. For each animal, the number of STEM121^+^ cells and cells co-expressing other markers were manually quantified using the Multi-point tool in FIJI. Percentages of marker-positive cells relative to STEM121^+^ cells were calculated. Graft volume was measured using Cavalieri’s method.^16^ Every 8th 40 µm section was analysed. Points intersecting STEM121^+^ graft areas were counted to estimate volume. All the quantifications were conducted with the experimenter blinded to the groups.

### Histological evaluation

Sections were stained with haematoxylin and eosin (H&E) to assess *in vivo* degradation of the hydrogel. The 40-µm-thick sections were washed with 1× PBS and mounted on glass slides with 0.5% gelatin. The sections were dehydrated in ethanol and stained with haematoxylin Gill’s (G2-2.5L, Amber Scientific, Australia) for 20 min. The sections were quickly dipped in 1% acid alcohol, then submerged in Scott’s Tap Water (SCOT-5L, Amber Scientific, Australia) for 30 s. They were rinsed with water, soaked in 95% ethanol for 20 s and then submerged in eosin (3801600, Leica Biosystems, Australia) for 3 min. The tissues were dehydrated and cleared with Xylene, then cover slipped with DPX and dried overnight before imaging under a brightfield microscope (Nikon TE2000E).

### Statistical analysis

The sample size was determined as the minimum number of animals required to achieve statistical significance at the 95% confidence level. *A priori* power analysis using G*power was conducted with an effect size of 2, based on previous data, an alpha error probability of 0.05, a desired power of 0.8 and two groups, which yielded a sample size of four animals per group. No criteria for exclusion were set and no animals were excluded from analysis.

Statistical analysis was performed in IBM SPSS Statistics (IBM Corporation, USA). Data were confirmed to be normally distributed using Levene’s test for equality of variances, and the independent *t*-test was used to compare treatment groups for differentiation markers and graft volumes. *P* < 0.05 was considered significant.

## Results

The survival and differentiation of hiLGEPs transplanted either in striatal differentiation medium or GelMA hydrogel were assessed 4 weeks following transplantation into the QA lesioned striatum. The GelMA hydrogel has a swelling ratio of 6.6 ± 0.1 and a Young’s modulus of 7.4 ± 0.196 kPa. Transplanted cells were identified with the human-specific cytoplasmic antibody, STEM121, co-expressed with the nuclei marker, DAPI. STEM121^+^/DAPI^+^ cells were detected in all animals, confirming survival of transplanted hiLGEPs in both delivery conditions (Fig. 3).

**Figure 3:**
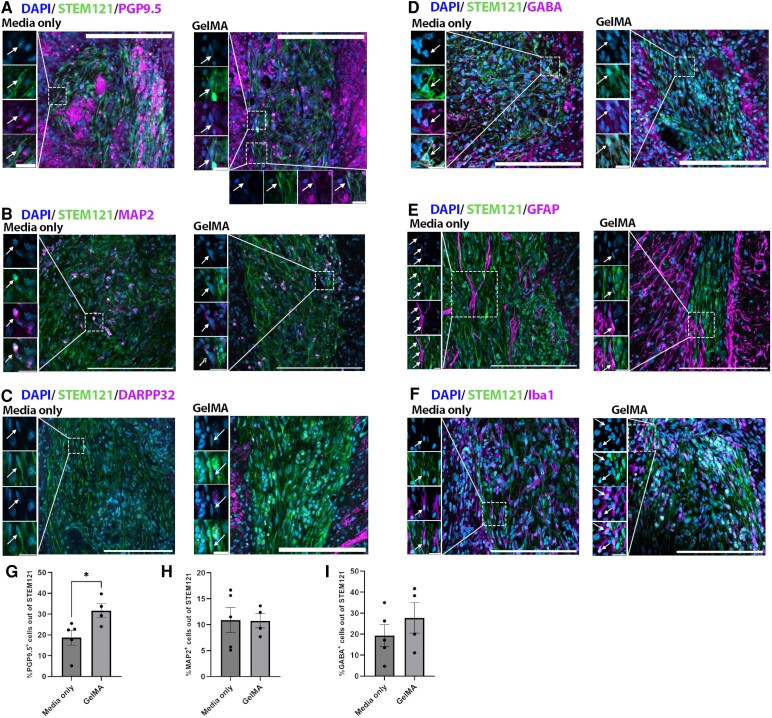
**GelMA-encapsulated hiLGEPs survive and differentiate within the lesioned striatum following *in vivo* transplantation.** The expression of (**A**) STEM121^+^/PGP9.5^+^, (**B**) STEM121^+^/MAP2^+^, (**C**) STEM121^+^/DARPP32^−^ and (**D**) STEM121^+^/GABA^+^ in hiLGEP-derived cells transplanted in media only or GelMA hydrogel at 4 weeks post-transplant. Expression of (**E**) STEM121^−^/GFAP^+^ and (**F**) STEM121^−^/Iba1^+^ cells in the transplanted brains at 4 weeks post-transplant. Scale bars 200 µm (large images, **A–F**) and 20 µm (high-magnification images, **A–F**). Graphs demonstrating the average percentage of (**G**) PGP9.5^+^/STEM121^+^ (*t*(7) = −2.581, *P* = 0.036), (**H**) MAP2^+^/STEM121^+^ (*t*(7) = 0.052; *P* = 0.960) and (**I**) GABA^+^/STEM121^+^ (*t*(7) = −0.966; *P* = 0.366) cells in media-only transplants (*n* = 5) or GelMA transplants (*n* = 4). Each individual data point represents the percentage of PGP9.5, MAP2 or GABA relative to STEM121 for each animal. Data represent mean ± SEM. Statistical significance was determined by an independent samples *t*-test, with * indicating *P* < 0.05.

To assess the effect of GelMA encapsulation on neuronal differentiation of hiLGEPs following transplantation, sections were co-stained with STEM121 and the neuronal marker PGP9.5 (Fig. 3A). hiLGEPs encapsulated in GelMA exhibited significantly higher neuronal differentiation, with 31.7% ± 3% PGP9.5^+^/STEM121^+^ cells compared with 18.7% ± 3.6% in the media-only transplant group (*t*(7) = −2.581; *P* = 0.036; Fig. 3G).

The maturation of transplanted hiLGEPs was evaluated through the expression of MAP2 (Fig. 3B). MAP2 staining revealed similar proportions of MAP2^+^/STEM121^+^ cells in both groups (media only: 10.9% ± 2.4%; GelMA: 10.8% ± 1.2%) with no significant difference between the groups (*t*(7) = 0.052; *P* = 0.960; Fig. 3H).

To confirm transplanted hiLGEPs differentiated to a striatal lineage, the expression of the medium spiny neuronal markers DARPP32 and GABA was examined (Fig. 3C and D). While no DARPP32^+^/STEM121^+^ cells were detected at 4 weeks post-transplant, GABA expression was detected in STEM121^+^ cells with a neuronal morphology in both transplant groups. The GelMA group showed 27.8% ± 6.6% GABA^+^/STEM121^+^ cells, whereas the media-only group showed 19.3% ± 5.2%. While higher in the GelMA group, this difference was not statistically significant (*t*(7) = −0.966; *P* = 0.366; Fig. 3I).

The presence of astrocytes and microglia was also investigated by GFAP and Iba1 expression, respectively (Fig. 3E and F). There were no GFAP^+^/STEM121^+^ cells present in either condition, indicating that GFAP^+^ cells were derived from the host (Fig. 3E). GFAP^+^ astrocytes were present around grafts in both groups. Interestingly, infiltration of host astrocytes into the graft core occurred in the media-only group but not in GelMA recipients, suggesting a barrier function of GelMA. Similarly, we observed expression of STEM121^−^/Iba1^+^ cells (Fig. 3F) within and around the graft core in both groups, confirming the presence of host-derived microglia.

Graft volumes were similar between groups (media only: 203.8 ± 28.1 µm^3^; GelMA: 186 ± 14.6 µm^3^; *t*(7) = 0.517; *P* = 0.621; Fig. 4A and B). This indicates that GelMA encapsulation did not significantly influence graft size. H&E analysis was used to assess hydrogel degradation. There was no evidence of residual GelMA hydrogel at the transplant site, suggesting that the matrix had degraded 4 weeks post-surgery (Fig. 4C).

**Figure 4:**
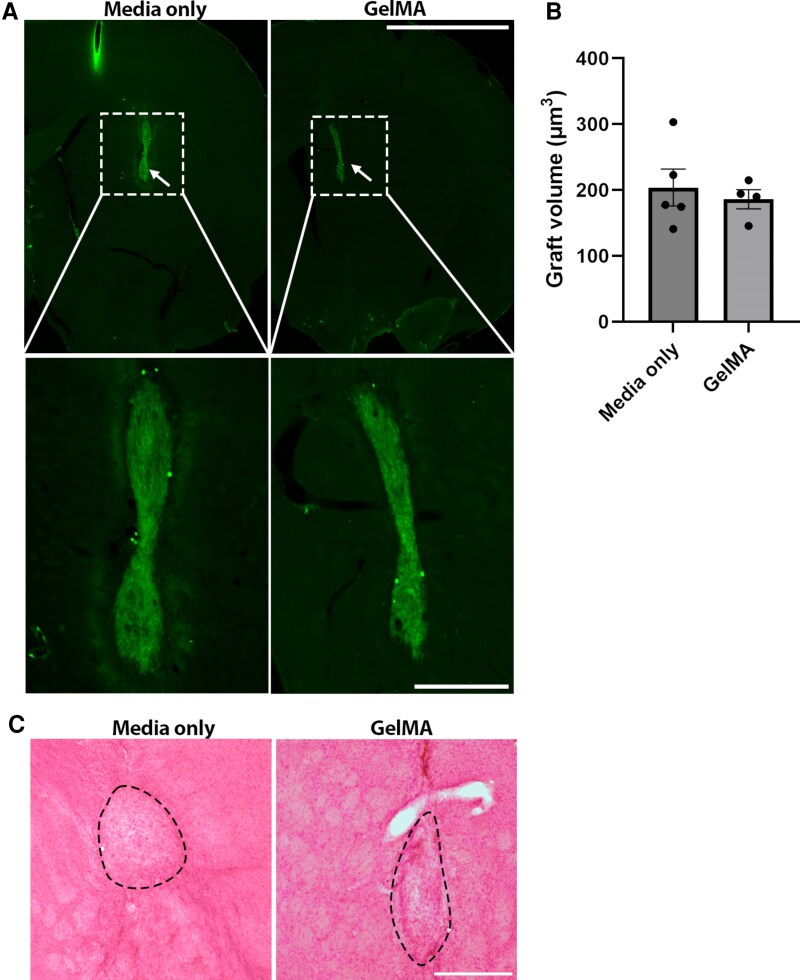
**Comparison of hiLGEP transplants in media only or GelMA hydrogel.** (**A**) STEM121^+^ grafts (white arrows) following cell transplantation into the striatum of QA-lesioned rats. Animals received hiLGEPs resuspended in either (i) media only or (ii) in GelMA hydrogel. Scale bar for the top panel is 3 mm, and the bottom panel is 500 µm. (**B**) Graph displaying the average graph volume for each group. Each individual data point represents the average graft volume for each animal. Data represent mean ± SEM with *n* = 4 for the media only group and *n* = 5 for the GelMA group. No significance was determined by an independent samples *t*-test (*t*(7) = 0.517; *P* = 0.621). (**C**) H&E-stained rat brain sections that were transplanted with cells resuspended in media only or in GelMA hydrogel. Black dotted lines indicate the transplant core. Scale bar 500 µm. GelMA, gelatin methacryloyl.

## Discussion

This study demonstrates that hiLGEPs encapsulated in GelMA hydrogel survive 4 weeks after transplantation into the QA-lesioned striatum and differentiated into a neuronal phenotype. The significantly greater proportion of PGP9.5^+^ neurons in GelMA-encapsulated grafts suggests that the hydrogel provides a supportive microenvironment that enhances early neuronal differentiation compared with cells delivered in media alone. STEM121^+^/GABA^+^ cells exhibiting a neuronal morphology were also detected at similar levels in both transplant groups. Consistent with our findings, PGP9.5 and GABA are both expressed in neurons at the early stage of nervous system development.^17-19^

MAP2 expression remained low in both transplant groups, indicating an immature neuronal status at 4 weeks post-transplant. MAP2, a cytoskeletal protein, is expressed at the early stage of neuronal differentiation, but the expression level correlates with cell maturity, with MAP2 upregulation and dendritic maturation occurring progressively over several weeks.^20,21^ Likewise, the absence of DARPP32^+^ MSNs is expected at this early stage; previous studies have shown DARPP32 expression emerges at ∼8 weeks post-transplant or later.^5,22-24^

Inflammatory responses were confirmed with the presence of GFAP^+^ astrocytes and Iba1^+^ microglia in the transplanted striatum; however, these glial cells were STEM121−, indicating they are derived from the host tissue in response to QA-induced lesioning and cell transplantation. STEM121^−^/Iba1^+^ cells are distributed throughout the transplant and surrounding the graft region for both conditions. This is consistent with their role as the resident immune surveillance cells, characterized by an immediate response to injury or tissue damage.^25^ In contrast, GFAP^+^ cells were absent within the GelMA grafts, suggesting that the GelMA hydrogel provides a protective interface against host astrocytic infiltration.

The comparable graft volumes observed across transplant groups indicate that the encapsulation does not impede graft establishment. To date, no studies have evaluated the graft volume of directly reprogrammed hiLGEPs; therefore, no direct comparison is currently possible. Most published work has used human pluripotent stem cell–derived neural stem cells for transplantation, with both the number of transplanted cells and the resulting graft volumes varying widely across studies.^22,24,26,27^ Collectively, these studies suggest that graft volume does not scale linearly with the number of transplanted cells, indicating that simply increasing the initial cell dose does not necessarily enhance graft survival. This non-linear relationship was previously examined in detail by Watts *et al*.^28^

Sprague–Dawley rats, an outbred strain, were used in this study. This means each rat has a diverse genetic background which renders them a good model as they appropriately mimic the genetic diversity of humans. With that said, the outbred status often leads to a high phenotypic and data variability which may lower the statistical power of experiments. It follows that this current study is a proof-of-concept study which should be replicated with larger cohort sizes and male and female rats to confirm the robustness of our findings.

In conclusion, this study demonstrates for the first time the capability to encapsulate directly reprogrammed hiLGEPs in GelMA hydrogel following transplantation into the striatum of a QA-lesioned rat model of Huntington’s disease. *In situ* visible-light crosslinking using an optical fibre enables hydrogel polymerization within brain tissue. Although soft matrices (0.1–1 kPa) are favourable for neuronal differentiation, as they mimic the extracellular matrix of brain tissue,^29^ the current study demonstrates that the stiffer substrate (7.4 kPa) can still support cell survival and differentiation *in vivo*. This could be attributed to two factors: firstly, the GelMA could have been softened over time due to enzymatic degradation *in vivo* after transplant, as supported by the absence of residual hydrogel 4 weeks post-transplant; and secondly, the arginyl-glycyl-aspartic acid peptide in the hydrogel enhances cell adhesion and provides crucial structural support during cell development *in vivo.*^10^ Overall, GelMA-encapsulated hiLGEPs survived transplantation and differentiated into early-stage neurons. GelMA also appeared to limit astrocytic infiltration.

These findings support GelMA hydrogel as a promising scaffold for CRT in Huntington’s disease. Further studies with longer experimental duration, multiple time points, larger cohorts and behavioural assessments are required to determine long-term maturation, integration and therapeutic efficacy. Since the QA-neurotoxic rodent model only provides an acute lesioning effect, a transgenic rodent model could be used in future studies to closely mimic the genetic profile and capture the progressive and complex nature of the disease. Lastly, future work should also replicate the study in female rats to investigate any gender effects on the results and to strengthen our conclusions that GelMA has potential as a scaffold for the transplantation of hiLGEPs.

## Data Availability

The data that support the findings of this study are available from the corresponding author upon reasonable request.

## References

[1] Vonsattel JP, Myers RH, Stevens TJ, Ferrante RJ, Bird ED, Richardson EP, Jr. Neuropathological classification of Huntington's disease. J Neuropathol Exp Neurol. 1985;44(6):559–577. 2932539 10.1097/00005072-198511000-00003

[2] Finkbeiner S . Huntington's disease. Cold Spring Harb Perspect Biol. 2011;3(6):a007476. 21441583 10.1101/cshperspect.a007476 PMC3098678

[3] Bunner KD, Rebec GV. Corticostriatal dysfunction in Huntington's disease: The basics. Front Hum Neurosci. 2016;10:317. 27445757 10.3389/fnhum.2016.00317 PMC4924423

[4] Rosser AE, Bachoud-Levi AC. Clinical trials of neural transplantation in Huntington's disease. Prog Brain Res. 2012;200:345–371. 23195427 10.1016/B978-0-444-59575-1.00016-8

[5] McCaughey-Chapman A, Burgers AL, Combrinck C, Marriott L, Gordon D, Connor B. Reprogrammed human lateral ganglionic eminence precursors generate striatal neurons and restore motor function in a rat model of Huntington's disease. Stem Cell Res Ther. 2024;15(1):448. 39578834 10.1186/s13287-024-04057-9 PMC11583420

[6] Foster AA, Marquardt LM, Heilshorn SC. The diverse roles of hydrogel mechanics in injectable stem cell transplantation. Curr Opin Chem Eng. 2017;15:15–23. 29085771 10.1016/j.coche.2016.11.003 PMC5659597

[7] Nguyen L, McCaughey-Chapman A, Connor B. GelMA hydrogel as a scaffold to enhance the survival and differentiation of human induced lateral ganglionic eminence precursor cells. J Neurosci Methods. 2024;405:110102. 38432304 10.1016/j.jneumeth.2024.110102

[8] Kobeissy FH, Hasan A, Al Rifai N, Gazalah H, Charara J. Culture of PC12 neuronal cells in GelMA hydrogel for brain tissue engineering. *2015 International Conference on Advances in Biomedical Engineering (ICABME)*. Beruit, Lebanon: Curran Associates, Inc. 2015:254-257.

[9] Liu Y, Yu H, Yu P, et al Gelatin methacryloyl hydrogel scaffold loaded with activated Schwann cells attenuates apoptosis and promotes functional recovery following spinal cord injury. Exp Ther Med. 2023;25(4):144. 36911380 10.3892/etm.2023.11843 PMC9995797

[10] Yue K, Trujillo-de Santiago G, Alvarez MM, Tamayol A, Annabi N, Khademhosseini A. Synthesis, properties, and biomedical applications of gelatin methacryloyl (GelMA) hydrogels. Biomaterials. 2015;73:254–271. 26414409 10.1016/j.biomaterials.2015.08.045 PMC4610009

[11] Bryant SJ, Nuttelman CR, Anseth KS. Cytocompatibility of UV and visible light photoinitiating systems on cultured NIH/3T3 fibroblasts in vitro. J Biomater Sci Polym Ed. 2000;11(5):439–457. 10896041 10.1163/156856200743805

[12] Noshadi I, Hong S, Sullivan KE, et al In vitro and in vivo analysis of visible light crosslinkable gelatin methacryloyl (GelMA) hydrogels. Biomater Sci. 2017;5(10):2093–2105. 28805830 10.1039/c7bm00110j PMC5614854

[13] Lim K, Schon BS, Mekhileri NV, et al New visible-light photoinitiating system for improved print fidelity in gelatin-based bioinks. ACS Biomater Sci Eng. 2016;2(10):1752–1762. 33440473 10.1021/acsbiomaterials.6b00149

[14] Lim KS, Klotz BJ, Lindberg GCJ, et al Visible light cross-linking of gelatin hydrogels offers an enhanced cell microenvironment with improved light penetration depth. Macromol Biosci. 2019;19(6):e1900098. 31026127 10.1002/mabi.201900098

[15] Chalard AE, Porritt H, Lam Po Tang EJ, et al Dynamic composite hydrogels of gelatin methacryloyl (GelMA) with supramolecular fibers for tissue engineering applications. Biomater Adv. 2024;163:213957. 39024864 10.1016/j.bioadv.2024.213957

[16] Altunkaynak BZ, Altunkaynak E, Unal D, Unal B. A novel application for the cavalieri principle: A stereological and methodological study. Eurasian J Med. 2009;41(2):99–101. 25610077 PMC4299836

[17] Schofield JN, Day IN, Thompson RJ, Edwards YH. PGP9.5, a ubiquitin C-terminal hydrolase; pattern of mRNA and protein expression during neural development in the mouse. Brain Res Dev Brain Res. 1995;85(2):229–238. 7600671 10.1016/0165-3806(94)00217-n

[18] Kent C, Clarke PJ. The immunolocalisation of the neuroendocrine specific protein PGP9.5 during neurogenesis in the rat. Brain Res Dev Brain Res. 1991;58(1):147–150. 1826643 10.1016/0165-3806(91)90248-h

[19] Wu C, Sun D. GABA receptors in brain development, function, and injury. Metab Brain Dis. 2015;30(2):367–379. 24820774 10.1007/s11011-014-9560-1 PMC4231020

[20] DeGiosio RA, Grubisha MJ, MacDonald ML, McKinney BC, Camacho CJ, Sweet RA. More than a marker: Potential pathogenic functions of MAP2. Front Mol Neurosci. 2022;15:974890. 36187353 10.3389/fnmol.2022.974890 PMC9525131

[21] Yuan X, Li W, Yan Q, Ou Y, Long Q, Zhang P. Biomarkers of mature neuronal differentiation and related diseases. Future Sci OA. 2024;10(1):2410146. 39429212 10.1080/20565623.2024.2410146 PMC11497955

[22] Arber C, Precious SV, Cambray S, et al Activin A directs striatal projection neuron differentiation of human pluripotent stem cells. Development. 2015;142(7):1375–1386. 25804741 10.1242/dev.117093 PMC4378247

[23] Besusso D, Schellino R, Boido M, et al Stem cell-derived human striatal progenitors innervate striatal targets and alleviate sensorimotor deficit in a rat model of Huntington disease. Stem Cell Reports. 2020;14(5):876–891. 32302555 10.1016/j.stemcr.2020.03.018 PMC7220987

[24] Schellino R, Besusso D, Parolisi R, et al hESC-derived striatal progenitors grafted into a Huntington's disease rat model support long-term functional motor recovery by differentiating, self-organizing and connecting into the lesioned striatum. Stem Cell Res Ther. 2023;14(1):189. 37507794 10.1186/s13287-023-03422-4 PMC10386300

[25] Kreutzberg GW . Microglia: A sensor for pathological events in the CNS. Trends Neurosci. 1996;19(8):312–318. 8843599 10.1016/0166-2236(96)10049-7

[26] Scaramuzza L, Ribodino M, Cassarino C, et al Transplanted human striatal progenitors exhibit functional integration and modulate host circuitry in a Huntington's disease animal model. Pharmacol Res. 2025;219:107905. 40796049 10.1016/j.phrs.2025.107905

[27] Choompoo N, Bartley OJM, Precious SV, et al Induced pluripotent stem cells derived from the developing striatum as a potential donor source for cell replacement therapy for Huntington disease. Cytotherapy. 2021;23(2):111–118. 33246883 10.1016/j.jcyt.2020.06.001 PMC7822401

[28] Watts C, McNamara IR, Dunnett SB. Volume and differentiation of striatal grafts in rats: Relationship to the number of cells implanted. Cell Transplant. 2000;9(1):65–72. 10784068 10.1177/096368970000900109

[29] Engler AJ, Sen S, Sweeney HL, Discher DE. Matrix elasticity directs stem cell lineage specification. Cell. 2006;126(4):677–689. 16923388 10.1016/j.cell.2006.06.044

